# A Targetless Method for Simultaneously Measuring Three-Degree-of-Freedom Angular Motion Errors with Digital Speckle Pattern Interferometry

**DOI:** 10.3390/s23073393

**Published:** 2023-03-23

**Authors:** Lili Shi, Sijin Wu, Miao Yan, Haisha Niu

**Affiliations:** 1 School of Instrumentation Science and Opto-Electronics Engineering, Beijing Information Science and Technology University, Beijing 100192, China; 2 Hopen Software Engineering Co., Ltd., Beijing 100192, China

**Keywords:** digital speckle pattern interferometry, three degrees of freedom, angular motion errors, pitch, yaw, roll

## Abstract

As a guide rail is the basic motion unit of precision equipment, the measurement of and compensation for its motion errors are important preconditions for precision machining and manufacturing. A targetless and simultaneous measurement method of three-degree-of-freedom (3-DOF) angular motion errors using digital speckle pattern interferometry (DSPI) is introduced in this paper. Based on the analysis of the sensitivity mechanism of DSPI to DOF errors and the formation mechanism of the phase fringes, the relationship between the angular motion errors and the distribution of the interferometric phases was established, and a new simultaneous measurement model of 3-DOF angular motion errors was further proposed. An optical setup based on a three-dimensional spatial-carrier DSPI with a right-angle symmetrical layout was used in the measurement system. Furthermore, repetitive tests, noise tests, and precision analysis were carried out to verify the performance of the system. The test results showed that the measurement resolution of the system was <1 μrad, which is capable of measuring the pitch angle, yaw angle, and roll angle at the submicron arc level simultaneously without target mirrors. The method has the advantages of no need to install cooperative targets and high measurement resolution, showing broad application prospects in many fields, including mechanical manufacturing, laser detection, aerospace, etc.

## 1. Introduction

As the advanced manufacturing industry develops by leaps and bounds, the demand for precision and ultra-precision machine tools is growing gradually and progressively. Computer numerical control (CNC) machine tools are the core production base of the equipment manufacturing industry, and there is an increasing demand for the tools’ precision requirements [1,2,3,4,5]. The precision detection of linear guide rails and rotary axes, which are the main precision motion units in the structure of CNC machine tools, is the key to precision machining and manufacturing [6]. Taking the linear guide rail as an example, its motion errors in three-dimensional (3D) space mainly consist of three linear errors along the axis directions (a positioning error in the direction of axis motion and two straightness errors perpendicular to the axis) and three angular errors of the motion around the axis (i.e., the pitch angle, yaw angle, and roll angle) [7,8]. The three angular errors are the key part of the six-degree-of-freedom (6-DOF) errors, and therefore, the research on the simultaneous measurement method of the three angular errors has become a topic of general interest in many fields, such as aerospace, mechanical manufacturing, and instrumentation. 

At present, the measurement methods for the pitch angle, yaw angle, and roll angle can be divided into optical measurement methods and non-optical measurement methods. The latter is mainly those using electronic levels [9], capacitive sensors [10,11], etc., most of which belong to contact measurements, with a small measurement range and cable connection required during work, resulting in limited application scenarios.

The research of optical measurement methods mostly concentrates on the laser multi-DOF measurement system [12,13,14], which can be divided into the laser collimation method, laser interferometry method, and combination method of laser collimation and laser interferometry. Sun et al. [15] proposed a simultaneous measurement method of three-degree-of-freedom (3-DOF) based on the autocollimation. On the basis of the traditional photoelectric autocollimator, a prism instead of a plane mirror was adopted as the cooperative target to achieve a spectral dimension ampliation measurement by coating on the front surface of the prism. The pitch and yaw angles were characterized by a slope of the right-angle prism, and the roll angle was characterized by two right-angle surfaces so as to realize the simultaneous measurement of the three angles. Although this method is simple in structure, in practical applications, the beam is susceptible to the influence of multiple error sources and the measurement precision of the system may be affected by the introduction of the cooperative prism. The laser interferometry method and combination method, such as the multi-axis laser interferometer [16], etc., enjoys the advantages of high measurement resolution and large measurement range. In the working process, it is required to install optical elements [17,18,19] (such as plane mirror, right-angle prism, grating, etc.) on the measured objects to reflect the laser beams to the detectors, and then calculate the angle values using optical information derived from the detectors. Liu et al. [20] put forward a simultaneous measurement system of long-distance 6-DOF geometric errors based on the laser interferometry, which allows for the simultaneous measurement of 3-DOF angular errors. The proposed measurement system, which combines the geometric optics with a laser interferometer, is characterized by less measuring time and a wider measuring range than traditional laser interferometers. Cui et al. [21] came up with a simultaneous measurement system of 6-DOF errors based on the combination of laser heterodyne interferometry and laser fiber collimation. Dual-frequency laser beams that are orthogonally linear polarized were adopted as the measuring datum. With the moving unit fixed to the measured axis by two cube-corner reflectors and a beam splitter, the measured unit receives the beams through a photodetector and carries out the photoelectric conversion to obtain the three angular errors. However, all of the above-mentioned measurement methods require the installation of light sources, detectors, or cooperative target mirrors on the objects to be measured. In terms of the installation of these cooperative targets, the influence of the self-weight and installation position of the equipment need to be taken into account since they may introduce additional errors to the measurement, which, as a result, leads to the limitation of their applications in many cases.

The digital speckle pattern interferometry (DSPI) technique—as a high-precision, non-contact and target-mirror-free optical measurement method—has been applied to the research of one-degree-of-freedom and two-degree-of-freedom angular motion measurements recently. For example, a roll angle measurement method based on DSPI was proposed [22]. By studying the relationship between the change in roll angle and the distribution of the interferometric phases of DSPI, the micro roll angle was successfully measured. In addition, a large-stroke roll angle measurement method was further proposed to solve the problem of the small single measurement stroke of the roll angle [23]. Later, the single-angle measurement and double-angle simultaneous measurement of the pitch angle and yaw angle were realized based on DSPI [24]. The outstanding advantages of DSPI in measuring the DOF of rigid bodies are demonstrated in the above research methods, that is, high-precision measurement without the need for cooperative target mirrors.

Because the online measurement of the geometric errors of the moving parts of the machine tool is a very time-consuming and frequent task, and the simultaneous measurement of multi-degree-of-freedom motion errors can greatly reduce the measurement time, it is of great significance to realize the simultaneous measurement of the 3-DOF angular motion errors. However, the simultaneous measurement of the 3-DOF angle motion errors using DSPI has not been realized yet. Due to the complexity of the measurement model and problems such as angle crosstalk, the aforementioned single-angle and double-angle measurement methods based on DSPI cannot be simply extended to the simultaneous measurement method of the three angles. In this article, a 3-DOF angular motion errors geometric measurement model based on DSPI is established, and a new simultaneous measurement method of 3-DOF angular motion errors is proposed. The proposed method can not only perform dynamic measurements with high measurement resolution without the cooperation of target mirrors but also can be applied to both the linear axis and rotary axis. The theoretical analysis and test results are both provided. The theoretical measurement resolution of the three angles is also discussed.

## 2. Principle

### 2.1. Geometric Model

**Figure 1:**
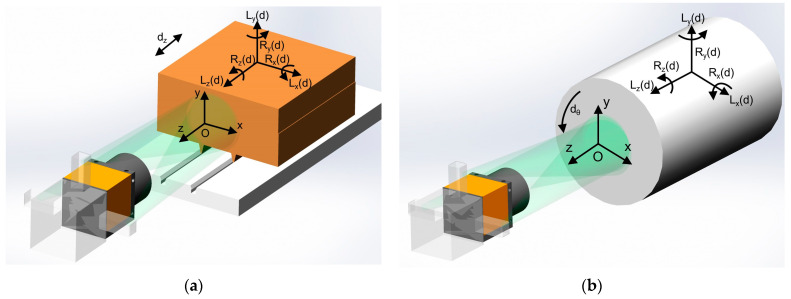
The setups of the DOF error measurement with DSPI for (**a**) linear guide and (**b**) rotary axis.

The motion parts in the equipment are divided into two types: the linear axis and the rotary axis. Their measurement setups are shown in Figure 1, where Figure 1a is the measurement setup of the linear axis and Figure 1b is that of the rotary axis. The three angular displacement errors around the *x*, *y*, and *z* directions are defined as $R_{x}\left(d\right)$, $R_{y}\left(d\right)$, and $R_{z}\left(d\right)$, respectively. Specifically, *d* refers to the linear displacement $d_{z}$ of the linear axis and the angular displacement $d_{\theta }$ of the rotary axis. In the DSPI measurement system, the linear displacement of the linear axis along the *z*-axis and the angular displacement of the rotary axis around the *z*-axis can be regarded as the accumulation of small displacement at a high sampling rate. Therefore, this measurement method is suitable for both the linear axis and the rotary axis.

**Figure 2:**
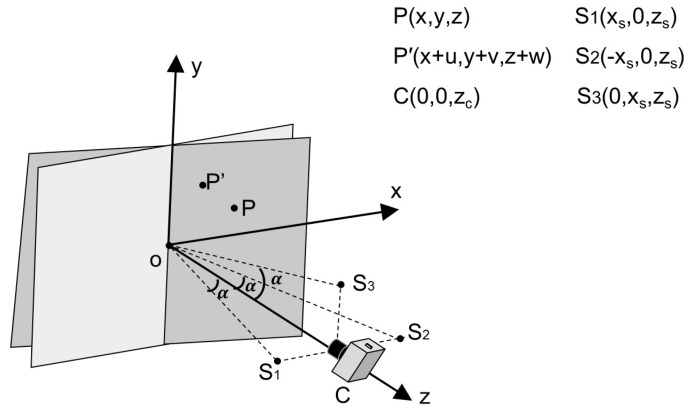
Geometric model of optical setups for measuring geometric motions of the rigid body by DPSI.

In the two measurement setups, the DSPI device is placed directly in front of the end face of the measured axis along the *z*-axis. The three laser beams from the upper, left, and right sides of the DSPI device are illuminated to the end face of the measured axis at a small angle. For the DSPI device, the motions of the linear axis or the rotary axis and the resulting 6-DOF errors belong to the geometric motions of the rigid body in the six degrees of freedom. Therefore, the above measurement settings can be simplified into a geometric model as shown in Figure 2. More specifically, camera C is on the *z*-axis, and the three lasers with the same wavelength, namely S1, S2, and S3, are symmetrically distributed on the upper, left, and right sides of the camera. The lasers irradiate the end face of the measured object and each laser beam interferes with its own reference beam at the camera to form three independent speckle pattern interferograms. The coordinate system and coordinate values of each point are shown in Figure 2.

When there are axis motion and 6-DOF errors at the same time, the arbitrary point P on the end face of the measured axis is transformed to point P′, and the coordinates change from (x,y,z) to (x+u,y+v,z+w). The three linear motions along the *x*, *y*, and *z* directions are defined as $L_{x}\left(d\right)$, $L_{y}\left(d\right)$, and $L_{z}\left(d\right)$, respectively, while the three angular motions rotating around the *x*, *y*, and *z* axes are $R_{x}\left(d\right)$, $R_{y}\left(d\right)$, and $R_{z}\left(d\right)$, respectively. As shown in Figure 2, the displacements generated in the *x*, *y*, and *z* directions are, respectively, represented by (u,v,w), where *u* is generated by $L_{x}\left(d\right)$, $R_{y}\left(d\right)$, and $R_{z}\left(d\right)$; *v* is generated by $L_{y}\left(d\right)$, $R_{x}\left(d\right)$, and $R_{z}\left(d\right)$; and *w* is generated by $L_{z}\left(d\right)$, $R_{x}\left(d\right)$, and $R_{y}\left(d\right)$. The expressions of *u*, *v*, and *w* are obtained as follows:

(1) $$\left(\begin{array}{c} u\\ v\\ w \end{array}\right)=\left(\begin{array}{c} L_{x}-x\left(1-\cos R_{y}\right)\;+\sqrt{x^{2}+y^{2}}\left[\cos\left(\arctan\frac{y}{x}+R_{z}\right)-\cos\left(\arctan\frac{y}{x}\right)\right]\\ L_{y}-y\left(1-\cos R_{x}\right)+\sqrt{x^{2}+y^{2}}\left[\sin\left(\arctan\frac{y}{x}+R_{z}\right)-\sin\left(\arctan\frac{y}{x}\right)\right]\\ L_{z}+\sqrt{y^{2}+z^{2}}\left[\sin\left(\arctan\frac{z}{y}+R_{x}\right)-\sin\left(\arctan\frac{z}{y}\right)\right]\;+\sqrt{x^{2}+z^{2}}\left[\sin\left(\arctan\frac{z}{x}-R_{y}\right)-\sin\left(\arctan\frac{z}{x}\right)\right] \end{array}\right).$$

Due to the small DOF errors, the higher-order terms of the DOF errors in Equation (1) can be ignored. Therefore, the equation is further simplified, as shown below:

(2) $$\left(\begin{array}{c} u\\ v\\ w \end{array}\right)=\left(\begin{array}{c} L_{x}-yR_{z}\\ L_{y}+xR_{z}\\ L_{z}+yR_{x}-xR_{y} \end{array}\right).$$

The relationship between the DOF errors and the geometric displacement is thereby established.

### 2.2. Measurement Principle

The geometric model shown in Figure 2 is composed of three independent and symmetrical digital speckle pattern interference optical paths with single-beam illumination, which are arranged symmetrically on the upper, left, and right sides of the optical axis. Each interference corresponds to an out-of-plane displacement component and an in-plane displacement component. In that case, the relationship between the distribution of the three interferometric phases and displacements is as follows [25]:

(3) $$\left(\begin{array}{c} \Delta \phi_{s1}\\ \Delta \phi_{s2}\\ \Delta \phi_{s3} \end{array}\right)=\frac{2\pi }{\lambda }\left(\begin{array}{c} u\sin\alpha +w\left(1+\cos\alpha \right)\\ u\sin\left(-\alpha \right)+w\left(1+\cos\alpha \right)\\ v\sin\alpha +w\left(1+\cos\alpha \right) \end{array}\right),$$

where, λ is the wavelength of the laser device and α is the illumination angle.

Considering the results shown in Equation (2), the phase increment output of the three interferences can be expressed as:

(4) $$\left(\begin{array}{c} \Delta \phi_{s1}\\ \Delta \phi_{s2}\\ \Delta \phi_{s3} \end{array}\right)=\frac{2\pi }{\lambda }\left(\begin{array}{c} L_{x}\sin\alpha +L_{z}\left(1+\cos\alpha \right)+yR_{x}\left(1+\cos\alpha \right)-xR_{y}\left(1+\cos\alpha \right)-yR_{z}\sin\alpha \\ -L_{x}\sin\alpha +L_{z}\left(1+\cos\alpha \right)+yR_{x}\left(1+\cos\alpha \right)-xR_{y}\left(1+\cos\alpha \right)+yR_{z}\sin\alpha \\ L_{y}\sin\alpha +L_{z}\left(1+\cos\alpha \right)+yR_{x}\left(1+\cos\alpha \right)-xR_{y}\left(1+\cos\alpha \right)+xR_{z}\sin\alpha \end{array}\right).$$

The phase of each interference is related to five geometric displacements, including two linear displacements and three angular displacements. When the illumination of the first and second interference optical paths is on the *xoz* plane, the interference optical path is sensitive to the remaining five geometric displacements, except for the $L_{y}$. Differently, when the illumination of the third interference optical path is on the *yoz* plane, the interference path is not sensitive to $L_{x}$. When there are one or more geometric displacements, the interferometric phases change accordingly, which explains the sensitivity of the DSPI to the DOF errors.

The derivatives of the three interferometric phases with respect to *x* and *y* are taken. Since the geometric displacement belongs to the rigid body displacements, with a spatial gradient of 0, the spatial gradients of the three interferometric phases along the *x* and *y* directions are expressed as:

(5) $$\left(\begin{array}{cc} \frac{\partial \Delta \phi_{s1}}{\partial x} & \frac{\partial \Delta \phi_{s1}}{\partial y}\\ \frac{\partial \Delta \phi_{s2}}{\partial x} & \frac{\partial \Delta \phi_{s2}}{\partial y}\\ \frac{\partial \Delta \phi_{s3}}{\partial x} & \frac{\partial \Delta \phi_{s3}}{\partial y} \end{array}\right)=\frac{2\pi }{\lambda }\left(\begin{array}{cc} -R_{y}\left(1+\cos\alpha \right) & R_{x}\left(1+\cos\alpha \right)-R_{z}\sin\alpha \\ -R_{y}\left(1+\cos\alpha \right) & R_{x}\left(1+\cos\alpha \right)+R_{z}\sin\alpha \;\\ -R_{y}\left(1+\cos\alpha \right)+R_{z}\sin\alpha & R_{x}\left(1+\cos\alpha \right)\;\; \end{array}\right).$$

It is revealed in Equation (5) that the spatial gradient of each digital speckle pattern interferometric phase is only related to one or two angular displacements, indicating that the generation of spatial fringes in the phase map is caused by the corresponding angular displacements only. When angular displacement variation occurs, the spatial gradient of the phases at a certain time is the same in the whole field, which suggests that the phase fringes are uniformly changed and the fringe spacing determined by the angular displacement between the two samplings is equal.

The expression of the three angular displacement errors can be obtained by solving part of the information in Equation (5). Considering that the spatial distribution of the phases varies uniformly in the whole field, the difference operation can be used to replace the differential operation, and the three angular displacement errors can be expressed as:

(6) $$\left(\begin{array}{c} R_{x}\left(d\right)\\ R_{y}\left(d\right)\\ R_{z}\left(d\right) \end{array}\right)=\frac{\lambda }{2\pi }\left(\begin{array}{c} \frac{\Delta \phi_{s3}\left(x_{1},y_{1},d\right)-\Delta \phi_{s3}\left(x_{2},y_{2},d\right)}{\left(1+\cos\alpha \right)\left(y_{1}-y_{2}\right)}\\ \frac{\Delta \phi_{s1}\left(x_{3},y_{3},d\right)-\Delta \phi_{s1}\left(x_{4},y_{4},d\right)}{\left(1+\cos\alpha \right)\left(x_{3}-x_{4}\right)}\\ \frac{\Delta \phi_{s2}\left(x_{5},y_{5},d\right)-\Delta \phi_{s2}\left(x_{6},y_{6},d\right)-\Delta \phi_{s1}\left(x_{5},y_{5},d\right)+\Delta \phi_{s1}\left(x_{6},y_{6},d\right)}{2\sin\alpha \left(y_{5}-y_{6}\right)} \end{array}\right),$$

where, $\left(x_{n},y_{n},d\right),\left(n\in N_{+}\right)$ stands for the spatial coordinates of the point, and *d* represents the displacement of the measured axis.

According to the above mathematical model, it can be seen that the 3-DOF angular motion errors are linearly related to the digital speckle pattern interferometric phases, and the measurement resolution of the 3-DOF angular motion mainly depends on the phase measurement resolution. Usually, in the measurement using DSPI, the phase measurement resolution can reach π/10, and be up to π/25 under optimal conditions. In general, with the phase resolution of π/10, the laser wavelength of 532 nm, and the illumination angle of 20°, the measurement resolution of the pitch, yaw, and roll angles can reach 0.14 μrad, 0.14 μrad, and 0.39 μrad, respectively, in theory when two symmetrical points about the origin with a distance of 100 mm are selected for analysis.

## 3. Results and Discussion

### 3.1. Experimental Setup

**Figure 3:**
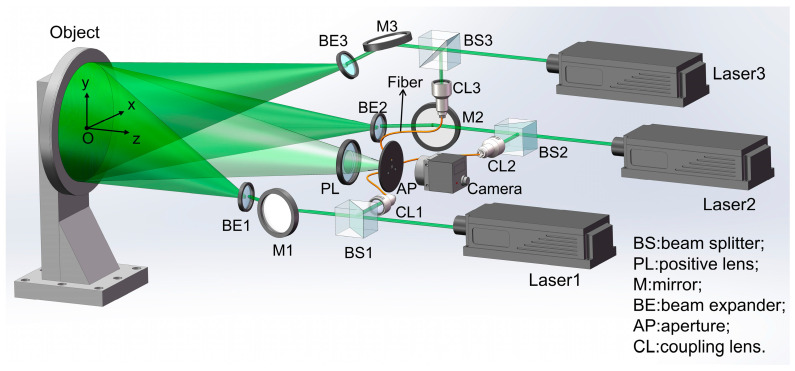
Optical setup of the simultaneous measurement system of 3-DOF angular motion errors using DSPI.

The measurement system proposed in this paper is shown in Figure 3. Three lasers with a central wavelength of 532 nm were utilized as the light source. A disc with a thickness of 1 mm and a radius of 32 mm was taken as the measured object, which was fixed to a six-axis piezoelectric oscillating table (Harbin Core Tomorrow Science & Technology Co., Ltd., Harbin, China, H63. XYZTR1S). An area of 13.4 mm × 13.4 mm in the center of the measured object was selected as the measurement area. By driving the oscillating table, tiny yaw, pitch, and roll motions were generated. The overall optical setup was composed of three interference parts, which had independent light sources and shared an imaging device and a detector. The illumination direction of the interference part containing Laser 3 was on the *yoz* plane, while the illumination directions of the other two interference parts were on the *xoz* plane. All illumination angles were set to about 26°. Taking the interference part where Laser 1 was located as an example, the laser beam was split into a reference beam and an object beam after passing through beam splitter 1 (BS1). The object beam was irradiated to the measured object after passing through mirror 1 (M1) and beam expander 1 (BE1), and then the diffusely reflected beam was collected by the imaging lens and captured by the camera through an aperture. The reference beam hit on the surface of the camera at a specific angle near the center of the aperture to interfere with the object beam after entering the optical fiber through the coupling lens 1 (CL1), yielding speckle pattern interferograms. When the geometric motion occurred on the measured disc, the optical path of the object beam changed while the optical path of the reference beam remained stable, thus changing the interferometric phase.

**Figure 4:**
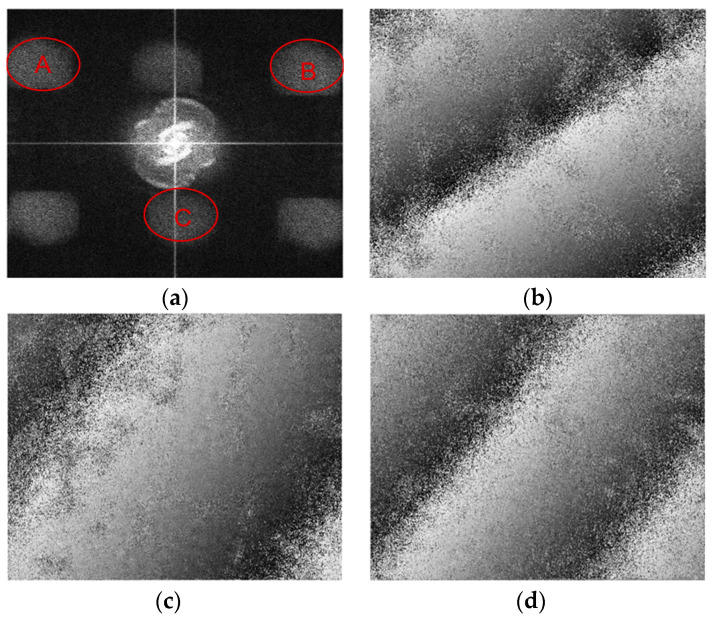
Frequency spectrum and phase maps: (**a**) spectrum obtained after Fourier transform; (**b**) wrapped phase map corresponding to frequency component A; (**c**) wrapped phase map corresponding to frequency component B; (**d**) wrapped phase map corresponding to frequency component C.

The three interferograms corresponding to the three interference parts were superimposed. By performing Fourier transform on the interferograms, the interferometric information was separated into three pairs of frequency components on the spectrum due to the difference in spatial-carrier frequencies of the three interference parts. Three halos, marked as A, B, and C in the spectrum, as shown in Figure 4a, were selected and used to generate three phase maps that are shown in Figure 4b–d by the means of inverse Fourier transform. These phase maps were derived from the three interference parts that contained Laser 1, Laser 2, and Laser 3, respectively.

After the phases shown in Figure 4 were unwrapped, several points at the edge of the phase maps were used to solve the pitch, yaw, and roll angles by performing the calculation shown in Equation (6). The results showed that the pitch, yaw, and roll angles were 10.11 μrad, 10.12 μrad, and 10.23 μrad, respectively, which were basically identical to the actual output values of the piezoelectric oscillating table (all of the three angles were nominally 10 μrad), verifying the feasibility of the method preliminarily.

### 3.2. Simultaneous Measurements of Pitch, Yaw, and Roll Angles

**Figure 5:**
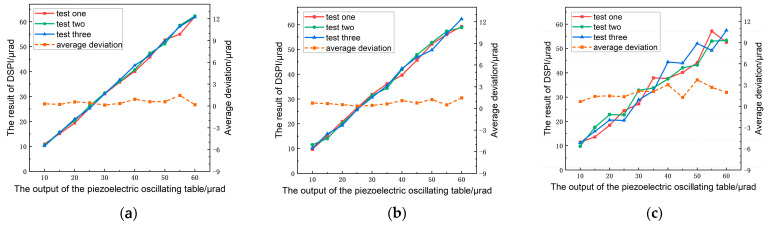
Simultaneous measurement results: (**a**) the measurement values of pitch, (**b**) the measurement values of yaw, (**c**) the measurement values of roll.

The pitch angle, yaw angle, and roll angle were simultaneously loaded by driving the six-axis piezoelectric oscillating table. The angle-loading ranges of the pitch angle, yaw angle, and roll angle set in the test were within 10–60 μrad, respectively. The three angle channels of the piezoelectric oscillating table were controlled to output the same angle value simultaneously, with a loading step of 5 μrad. Finally, a total of 11 data points were obtained, and the measurement process was repeated three times to obtain three sets of repeated test data. The measurement results and average deviation are shown in Figure 5.

The coincidence of the three groups of tests can be obviously reflected by the values shown in Figure 5. In particular, the coincidence of the measurement curves of the pitch and yaw angles was very good, while the coincidence of the roll angle data was slightly poor, but the fluctuation was within the acceptable limits. Under different measurement steps, the average deviation of pitch ranged from 0.14 μrad to 1.45 μrad, with an average relative error of 3.54% compared with the piezoelectric oscillating table. The average deviation of yaw was within the range of 0.33–1.45 μrad, with an average relative error of 3.71%. The average deviation of roll was within the range of 0.68–3.72 μrad, with an average relative error of 8.32%.

The close-loop piezoelectric oscillating table used in the experiment has good linearity and repeatability accuracy, reaching 0.25% F.S. and 0.2% F.S., respectively, with a maximum stroke of 600 μrad per axis. The closeness of the three-angle measurement results to the outputs of the piezoelectric oscillating table indicates that the proposed method has high accuracy in measurement, and proves that this method can reliably realize the simultaneous measurement of the 3-DOF angular motion errors.

### 3.3. Repeatability Test

**Figure 6:**
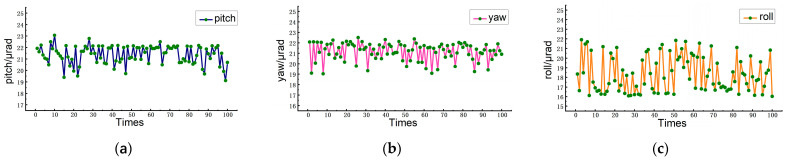
Repeatability experiment results: (**a**) results from pitch loading, (**b**) results from yaw loading, (**c**) results from roll loading.

Aiming to verify the measurement reliability of the proposed measurement system, the pitch angle, yaw angle, and roll angle were measured repeatedly 100 times with a loading angle of 20 μrad by the piezoelectric oscillating table. The measurement results are shown in Figure 6. It can be seen that the measured pitch angle fluctuated within the range of 19.11–23.06 μrad, with an average of 21.39 μrad and a standard deviation of 0.81 μrad. The measured yaw angle fluctuated between 19.03 μrad and 22.50 μrad with an average of 21.16 μrad and a standard deviation of 0.85 μrad. The measured roll angle fluctuated between 16.03 μrad and 21.91 μrad with an average of 18.33 μrad and a standard deviation of 1.82 μrad. The low standard deviation of the measurement values of the three angles verified the high repeatability of the overall measurement. The factors affecting the fluctuation of the measurement results of the three angles mainly include phase noise, environmental disturbance, etc.

### 3.4. Noise Evaluation

**Figure 7:**
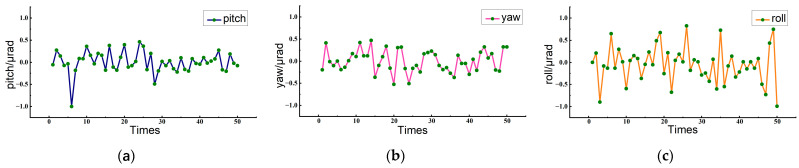
Noise evaluation experiment: (**a**) measurement results of pitch, (**b**) measurement results of yaw, (**c**) measurement results of roll.

In order to verify the influence of random errors caused by noise interference on the measurement system, measurements were carried out 50 times, with the original measurement system remaining unchanged and the measured object remaining stationary. The data set obtained by DSPI is exhibited in Figure 7.

The data in Figure 7 show that when the object was stationary, phase noise was detected at all three angles, of which the standard deviations were 0.238 μrad, 0.242 μrad, and 0.403 μrad, respectively. If twice the standard deviation was taken as the noise value, the three-angle noise values were 0.476 μrad, 0.484 μrad, and 0.806 μrad, respectively, which were all less than 1 μrad and close to the theoretical measurement resolution. It is verified that the DSPI measurement system has a high resolution and can be used for the simultaneous measurement of the pitch angle, yaw angle, and roll angle at the submicron arc level. During the test, the measurement resolution can be further improved and optimized by adopting a shorter wavelength laser source, and especially, expanding the measurement area. In this test, the measurement area was 13.4 mm × 13.4 mm, so the distance between the two selected points was short. If the measurement area is larger and two points at a greater distance can be selected for calculating the angular motion errors, then the measurement resolution will be improved.

The experimental results show that the measurement performance of the roll angle is weaker than that of the pitch angle and yaw angle. This is consistent with theoretical analysis. As can be seen from Equation (6), both the pitch and yaw angles can be solved from one phase map, while the roll angle requires two phase maps to solve. This shows that the measurement accuracy of the roll angle is more susceptible to phase errors. Moreover, roll angle measurement has a higher sensitivity coefficient. In these experiments, the illumination angles were 26°, so the sensitivity coefficient of roll measurement was about twice those of pitch angle and yaw angle measurements. These explain why roll angles were slightly less measured. However, the measurement of the roll angle as well as the measurements of pitch and yaw angles is a high-precision measurement method.

## 4. Conclusions

A simultaneous measurement method of 3-DOF angular motion errors based on DSPI is proposed. The method does not require any cooperative target and does not require any pretreatment of the measured object, which is a true non-contact, pollution-free measurement method. It not only eliminates the inconvenience and measurement errors caused by the installation of the target mirrors, but also provides a feasible solution for applications where the target mirror cannot be installed. Because this method belongs to the optical interference method, its measurement resolution and measurement accuracy are very high. Moreover, compared with the traditional optical interference methods of 3-DOF angular motion error measurement, in addition to eliminating the need for a cooperative target, the optical setup of this method is simple, easy to integrate, and highly reliable. This method mainly relies on the diffused light from the rough surface of the measured object for interference. Therefore, in the field of engineering, it can be applied to most measured objects except for objects with a specular surface.

In this article, the measuring principle of the proposed method is described in detail, and the performance of the measurement method is also demonstrated through theoretical analysis and experimentation. It can be concluded from the theoretical analysis and experimental results that this measurement method has an edge in terms of precision, requiring no target mirrors, and is applicable to both the linear axis and the rotary axis. Therefore, it has a certain application prospect in rigid body angle measurement and other fields, laying a foundation for the research of the simultaneous measurement of 6-DOF errors.

## Data Availability

Data are available from the corresponding authors on reasonable request.
